# Status of Biofilm-Forming Genes among Jordanian Nasal Carriers of Methicillin-Sensitive and Methicillin-Resistant *Staphylococcus aureus*

**DOI:** 10.29252/ibj.24.6.381

**Published:** 2020-07-08

**Authors:** Ashraf I Khasawneh, Nisreen Himsawi, Jumana Abu-Raideh, Muna A. Salameh, Mohammad Al-Tamimi, Sameer Al Haj Mahmoud, Tareq Saleh

**Affiliations:** 1 Department of Basic Medical Sciences, Faculty of Medicine, Hashemite University, Zarqa, Jordan;; 2 Department of Basic Medical Sciences, College of Medicine, Al-Balqa' Applied University, Al-Salt, Jordan

**Keywords:** Biofilms, Methicillin-resistant *Staphylococcus aureus*, Methicillin-sensitive *Staphylococcus aureus*

## Abstract

**Introduction:**

Biofilm formation in *Staphylococcus* *aureus* is a major virulence factor. Both MSSA and MRSA are common causes of community- and hospital-acquired infections and are associated with biofilm formation. The status of biofilm-forming genes has not been explored in Jordanian nasal carriers of *S. aureus*. This study investigates antibiotic resistance patterns and the prevalence of biofilm-forming genes between MSSA and MRSA in two distinct populations in Jordan.

**Methods:**

A total of 35 MSSA and 22 MRSA isolates were recovered from hospitalized patients and medical students at Prince Hamzah Hospital, Jordan. Antibiotic susceptibility was tested using disk diffusion method and Vitek 2 system. The phenotypic biofilm formation was tested using CRA and microtiter plate assays. The prevalence of the biofilm-forming genes was determined using multiplex PCR.

**Results:**

Among 57 *S.* *aureus* isolates, 22 (38.6%) isolates were MRSA and were highly resistant against benzylpenicillin, oxacillin, and imipenem. The frequencies of the *icaADBC* were 77.1%, 97.1%, 94.3%, and 97.1% respectively in MSSA compared to 86.4%, 100%, 100%, and 100% in MRSA isolates. On the other hand, the frequency of the *fnbA*, *fnbB*, *clfA*, *fib*, *clfB*, *ebps*, *eno*, and *cna* genes was 81.8%, 90.9%, 95.5%, 90.9%, 86.4%, 100%, 100%, and 40.9%, respectively in the MRSA isolates.

**Conclusion:**

In both groups, MRSA isolates, in comparison to MSSA, were significantly more resistant to cefoxitin, oxacillin, imipenem, tetracycline, clindamycin, and trimethoprim-sulfamethoxazole. Unexpectedly, biofilm formation and gene prevalence between MRSA and MSSA isolates showed no significant difference, suggesting other potential virulence mechanisms.

## INTRODUCTION

*Staphylococcus aureus* is a main component of the human normal flora of the skin, mucosal surfaces, and the nasal cavity. Nasal carriers of *S. aureus* might develop an endogenous infection, and consequently, they pose a threat of transmitting infection to susceptible individuals^[^^1^^]^. Accordingly, *S. aureus* accounts for the high numbers of nosocomial and community-acquired infections, as well as biomaterial contamination^[^^2^^,^^3^^]^. Moreover, these microorganisms have the potential to colonize medical catheters, tubing, surgical drains, and central lines by forming biofilms, which render them resistant to host defense mechanisms and antimicrobial therapy^[^^4^^,^^5^^]^. 

*S. aureus* is divided into two main categories: MSSA and MRSA, based on their sensitivity to the *β*-lactam, methicillin. MRSA infections have surged dramatically in the past 10-15 years and are increasingly becoming a major source of nosocomial infections that are associated with high morbidity and mortality^[^^6^^]^. Several mechanisms have been identified to be involved in the development of resistance in *Staphylococcus* species^[^^7^^]^. Among these mechanisms, the biofilm formation is considered as a major contributor to antibiotic resistance. Interestingly, both MRSA and MSSA has the ability to form biofilms through a polysaccharide intracellular adhesion and MSCRAMMs^[^^8^^,^^9^^]^. These molecules play an important role in initiating attachment to bone, joints, endovascular tissue, and biomaterials^[^^10^^]^. Polysaccharide intracellular adhesion encoded by *ica*ADBC locus has been indicated to be required for biofilm production in staphylococci^[^^11^^]^. It has further been shown that the co-expression of *icaA* and *icaD* arbitrates the synthesis of sugar oligomers and also the expression of the capsular polysaccharide using the enzyme N-acetylglucosaminyltransferase^[^^12^^]^. Furthermore, *S. aureus *species MSCRAMM molecules are covalently linked to the cell wall by sortase via the LPXTG motif, and include the following proteins: *clfA* and *clfB*, *fib*, *fnbA* and *fnbB*, *cna* (collagen-binding protein), *ebps*, and *eno* (laminin-binding protein)^[^^13^^]^. 

Despite similarity, MRSA and MSSA may express different levels of the biofilm-forming genes, especially when both types of strains show different sensitivity profiles to a wide array of antibiotics^[^^14^^]^. Unfortunately, a comprehensive analysis of antibiotic resistance pattern, biofilm formation, as well as prevalence of biofilm-forming genes between MSSA and MRSA has not been carried out in Jordanian carriers before. Thus, in this work, we aim to compare the prevalence of biofilm-related genes and their ability to form biofilm between MRSA and MSSA isolates from two cohorts of Jordanian *S. aureus* carriers, representing a group of patients admitted to a central hospital and medical students under training in the same institution.

## MATERIALS AND METHODS

**Study participants**

In this cross-sectional study, 202 inpatients at Prince Hamzah Hospital, Amman, Jordan, and 290 medical students at the Faculty of Medicine, the Hashemite University, Zarqa, Jordan were recruited voluntarily from June 2015 to August 2017. All participants consented to nasal swab collection and were invited to complete a questionnaire designed to comprehensively review the demographic information for every participant. The demographic data of both populations are illustrated in Tables 1 and 2 and in our previous publication^[^^15^^]^. 

**Nasal swab collection**

The collection of nasal swabs was carried out as described before^[^^15^^]^. Briefly, samples were collected under aseptic conditions, and the same swab was used for both nostrils. Swabs were labeled appropriately and transferred to the microbiology laboratory, in an icebox within one hour.

**Identification of** ***S. aureus***

Nasal swabs were cultured on a nutrient agar (Mast, UK), sheep blood agar (Mast), and mannitol salt agar (Biolab, Hungary) and incubated at 35 °C for 24 hours. Positive cultures were assessed for colony morphology, Gram stain (GCC Diagnostics, UK), and biochemical tests. Isolates were diagnosed as *S. aureus* if they had a typical Gram-positive cocci shape arranged in clusters and tested positive for catalase and coagulase enzymes, as well as formed yellow colonies on mannitol salt agar and creamy colonies on sheep blood agar (Biolife, Italy)^[^^15^^]^. All positive isolates were further analyzed using the automatic Vitek 2 compact system for Gram-positive identification (GP card, REF 21342) according to manufacturer’s instructions. The strains used as a quality control were as follows: ATCC^®^ 33591 (MRSA) and ATCC^®^ 25923 (MSSA).

**CRA assay**

CRA plates were prepared by adding 0.8 g/L Congo red dye (VWR Prolabo, USA) and 36 g/L sucrose (GCC Diagnostics) to 1 L of Brain Heart Infusion (Biolab Zrt, Hungary). Isolates were cultured on CRA plates and incubated aerobically in triplicates at 37 ºC for 24 h. Black colonies represented slime-producing isolates, while red colonies represented non-slime-producing isolates.

**Microtiter plate biofilm assay**

Controls and isolates were incubated in Trypticase soy broth (Biolab Zrt) overnight and then diluted 100fold in the mentioned broth containing 2% (w/v) glucose. Subsequently, 200 µL of the diluted cultures was transferred to the wells of a sterile 96-well polystyrene plate and incubated at 37 ºC for 24 h. The content of wells was then discarded and washed three times with phosphate-buffered saline to remove non-adherent cells, followed by an air-drying step for fixation. After that, the wells were stained with 1% CV (GCC Diagnostics) at room temperature for 5 minutes and then washed three times with sterile distilled water and air-dried. The bound CV was dissolved in 95% ethanol, and the level of CV was determined by measuring the OD using a spectrophotometer (Tecan Life Science, Switzerland) at the wavelength of 570 nm. The quantitative biofilm assay was performed in quadruple for all isolates and controls, and the assay was repeated three times. The OD cut-off value was calculated as the mean of the negative control strain plus three standard deviations. This value was used to categorize the biofilm formation abilities of different isolates. The isolates were considered as non-biofilm producers when the OD value was <0.2, weak biofilm producer when the OD value was between 0.2 and 0.4, moderate biofilm producer when the OD value was between 0.4 and 0.8, and strong biofilm producer when the OD value was >0.8^[^^16^^]^.

**Table 1:** Association of demographic and clinical characteristics with MRSA and MSSA colonization among Prince Hamza Hospital participants

**Sociodemographics and clinical characteristics**	**N (%)**	**MRSA**			**MSSA**		
		**N (%)**	***p*** ** value**	**Odds ratio of having MRSA** **(95% CI)**	**N (%)**	***p*** ** value**	**Odds ratio of having MSSA (95% CI)**
**Age** <25 25-50 51-75 >75	23 (11.4) 79 (39.1) 86 (42.6) 14 (6.9)	1 (4.3) 5 (6.3) 5 (5.8) 0 (0.0)	0.801		1 (4.3) 6 (7.6) 7 (8.1) 2 (14)	0.754	
							
**Gender** Male Female	82 (40.6) 120 (59.4)	4 (4.9) 7 (5.8)	0.769	0.828 (0.234-2.924)	6 (7.3) 10 (8.3)	0.793	0.868 (0.303-2.490)
							
**Hospital ward** Medicine Surgery Orthopedic Gynecology	69 (34.2) 60 (29.7) 34 (16.8) 39 (19.3)	6 (8.7) 4 (6.6) 1 (2.9) 0 (0.0)	0.236		6 (8.7) 3 (5.0) 4 (11.7) 3 (7.7)	0.694	
							
**Smoking** Yes No	65 (32.2) 137 (67.8)	4 (6.2) 7 (5.1)	0.760	1.218 (0.344-4.317)	4 (6.2) 12 (8.8)	0.522	0.683 (0.212-2.206)
							
**Hospital admission** Yes No	137 (67.8) 65 (32.2)	7 (7.1) 4 (6.2)	0.760	0.821 (0.232-2.911)	10 (7.3) 6 (9.2)	0.635	0.774 (0.269-2.231)
							
**Previous surgeries** Yes No	118 (58.4) 84 (41.6)	8 (6.8) 3 (3.6)	0.322	1.964 (0.505-7.632)	10 (8.5) 6 (7.1)	0.730	1.204 (0.420-3.451)
							
**Chronic disease** Yes No	95 (47) 107 (53)	8 (8.4) 3 (2.8)	0.079	3.188 (0.821-12.384)	8 (8.4) 8 (7.5)	0.804	1.138 (0.410-3.160)
							
**Antibiotic use in the past two weeks** Yes No	98 (48.5) 104 (51.5)	6 (6.1) 5 (4.8)	0.681	1.291 (0.381-4.375)	6 (6.1) 10 (9.6)	0.358	0.613 (0.214-1.756)
							
**URTI symptoms** Yes No	17 (8.4) 185(91.6)	2 (11.8) 9 (4.9)	0.230	2.607 (0.516-13.180)	2 (11.8) 14 (7.6)	0.540	1.629 (0.338-7.850)

**Table 2:** Association of demographic and clinical characteristics with MRSA and MSSA colonization among medical student participants

** Sociodemographics and clinical characteristics**		**N (%)**	**MRSA**			**MSSA**		
			**N (%)**	***p*** **value**	**Odds ratio of ** **having MRSA ** **(95% CI)**	**N (%)**	***p*** **value**	**Odds ratio of having MSSA (95% CI)**
**Age** Years: 19.7 ± 2		290 (100)	9 (3.1)	0.855		21 (7.2)	0.026	
								
**Gender** Male Female		179 (61.7) 111 (38.3)	5 (2.8) 4 (3.6)	0.699	0.769 (0.202-2.926)	13 (7.3) 8 (7.2)	**0.986 **	1.008 (0.404- 2.516)
								
**Year of medical school** First year Second year Third year Fourth year		80 (27.6) 52 (18.0) 57 (19.6) 101 (34.8)	0 (0.0) 3 (33.3) 1 (11.1) 5 (55.6)	0.152	2 (2.5) 0 (0.0) 4 (7.0) 15 (14.9)		**0.001**	
								
**Smoking** Yes No		68 (23.4) 222 (76.6)	2 (2.9) 7 (3.2)	0.930	0.931 (0.189-4.589)	4 (5.9) 17 (7.7)	0.621	0.754 (0.245-2.321)
								
**Work or training in hospital** Yes No		103 (35.5) 187 (64.5)	4 (3.9) 5 (2.7)	0.570	1.471 (0.386-5.602)	16 (15.5) 5 (2.7)	**0.001**	**6.809** **(2.416-19.195)**
								
**Previous hospitalization admission** Yes No		75 (25.9) 215 (74.1)	1 (1.3) 8 (3.7)	0.354	0.384 (0.047-3.125)	5 (6.7) 16 (7.4)	0.971	0.981 (0.346-2.781)
								
**Previous surgeries** Yes No		64 (22.0) 226 (78.0)	2 (3.1) 7 (3.1)	0.950	1.052 (0.213-5.198)	3 (4.7) 18 (8.0)	0.410	0.593 (0.169-2.083)
								
**Chronic disease** Yes No		18 (6.2) 272 (93.8)	2 (11.1) 7 (2.6)	0.034	5.067 (0.968-26.522)	1 (5.6) 20 (7.4)	0.824	0.791 (0.100-6.271
								
**Antibiotic use in the past two weeks** Yes No		24 (8.3) 266 (91.7)	0 (0.0) 9 (3.4)	0.750	0.711 (0.087-5.835)	3 (12.5) 18 (6.8)	0.263	2.075 (0.563-7.647)
								
**URTI symptoms** Yes No		24 (8.3) 266 (91.7)	1 (2.3) 8 (3.3)	0.084	5.729 (0.616-53.316)	1 (2.3) 20 (8.1)	0.178	0.270 (0.035-2.068)
								

**Antibiotic susceptibility tests**

Antibiotic susceptibility testing using the standard disk diffusion method was carried out on Mueller-Hinton Agar (Liofilchem, Italy) according to the latest CLSI guidelines (Clinical and Laboratory Standards Institute, performance standards for antimicrobial susceptibility testing, 28^th^ informational supplement Jan 2018). The antibiotics used were oxacillin (1 µg), cefoxitin (30 µg), and vancomycin (30 µg; Bioanalyse, UK). Antibiotic susceptibility patterns were also determined and analyzed by Vitek 2 compact system using the Gram-positive *susceptibility card (AST P592, REF 22287) according to the manufacturer’s recommendation (BioMérieux, France)^[^^15^^]^.

**Multiplex PCR detection of biofilm genes**

Bacterial DNA was extracted from culture samples, using a DNA extraction kit (Qiagen, Hilden, Germany) following the protocol for Gram-positive bacteria as instructed by the manufacturer. Primers used for the identification of methicillin resistance gene (*mecA*) and those used for the detection of 12 biofilm genes (*icaA*, *icaB*, *icaC*, *icaD*, *CIfA*,* CIfB*, *cna*, *ebps*, *eno*, *fib*, *fnbA*, and *fnbB*) were obtained from Princess Haya Biotechnology Center at Jordan University of Science and Technology^[^^17^^]^. PCR amplification for *mecA* gene was carried out on a GenePro Thermal Cycler (Bioer Technology Co., Ltd., China) using the following steps: initial denaturation at 95 ºC for 5 min, followed by 30 cycles at 94 ºC for 2 min, 57 ºC for 2 min, and 72 ºC for 1 min, with a final extension step of 72 ºC for 7 min^[^^18^^]^. Multiplex PCR was carried out for the biofilm genes using Qiagen Multiplex PCR Kit based on the following thermal steps: initial denaturation at 95 ºC for 15 min, followed by 35 cycles at 94 ºC for 30 s, 60 ºC for 90 s, and 72 ºC for 90 s, and a final extension step at 72 ºC for 10 min^[^^17^^]^. PCR products were electrophoresed on a 1.5% agarose gel stained with ethidium bromide and visualized using UV transillumination (Alpha Innotech, USA).

**Statistical analysis**

Statistical analysis was performed using SPSS version 22. Differences in proportions were tested by Chi-square test. *p* values less than 0.05 were considered statistically significant.

**Ethical statement**

The above-mentioned sampling protocols were approved by the Institutional Review Board (IRB) of the Hashemite University and Prince Hamzah hospital (ethical code: 1/8/2013/2014 and MH/32/2047 respectively). Written informed consents were provided by all the participants.

## RESULTS

**Characteristics of study population**

The study population was divided into two groups: Patient group comprising 202 participants and the student group comprising 290 participants. Recruited patients were hospitalized in Prince Hamzah Hospital in the following clinical wards: Internal Medicine, General Surgery, Orthopedic, and Gynecology. The mean age of the patient group was 50 years old. Females constituted 59.4% of the patients, while 40.6% were males. Among these, 32.2% were smokers. Further details, including hospital admissions, previous surgeries, chronic illnesses, antibiotic usage, previous respiratory tract infections, and history of fever are detailed in Table 1. The student group included students in the preclinical (65.2%) and clinical training levels (34.8%). Their mean age was 19.7 years, and males represented 61.7% of the participants. Past medical and social history data for the medical students are listed in Table 2. 

***S. aureus*** **d** **istribution**

First,* S. aureus* isolates were identified in 57 (11.6%) out of 492 nasal swabs collected from both patients and students. Using phenotypic (disc diffusion) method, Vitek 2 system, and genotyping (PCR for *mec A*), we were able to confirm that 22 (38.6%) out of the 57 *S. aureus*-positive isolates were MRSA (data not shown). In the patient group, 5.4% of the *S. aureus*-positive isolates were MRSA and 7.9% were MSSA, while in the student group, only 3.1% of the *S. aureus*-positive samples were MRSA and 7.2% were MSSA (Tables 1 and 2). Amongst patients, the majority of *S. aureus*-positive isolates (50%) originated from the samples collected from the Internal Medicine ward (Table 1). However, there was no statistically significant relationship between the nasal isolation of *S. aureus* and the age, gender, or department of admission of the participating patients (Table 1). Conversely, the age of the participating medical students, level, and hospital training appear to significantly affect the distribution of *S. aureus *strains (Table 2). For instance, students older than 21 years old who were in the clinical education level and had started their hospital training had higher MSSA infection rates in comparison to their younger counterparts who have not started their clinical training yet (Table 2). In addition, medical students starting their clinical training were more susceptible to nosocomial* S. aureus *infection. 

**Biofilm assay on CRA**

After the potential of biofilm production by MRSA and MSSA isolates was examined, the isolates were inoculated onto CRA and followed up for 72 hours to detect biofilm formation. Red-colored colonies indicated non-biofilm forming isolates, while black-colored colonies represented biofilm-forming isolates. MRSA isolates showed a 90.9% (20/22) capability of biofilm production, consistent with the findings reported elsewhere^[^^19^^,^^20^^]^. Interestingly, only 25/35 (71.4%) of the MSSA isolates were capable of biofilm production, suggesting strain variability of biofilm regulatory gene production and the associated surface proteins.

**Microtiter plate assay**

**Fig. 1:**
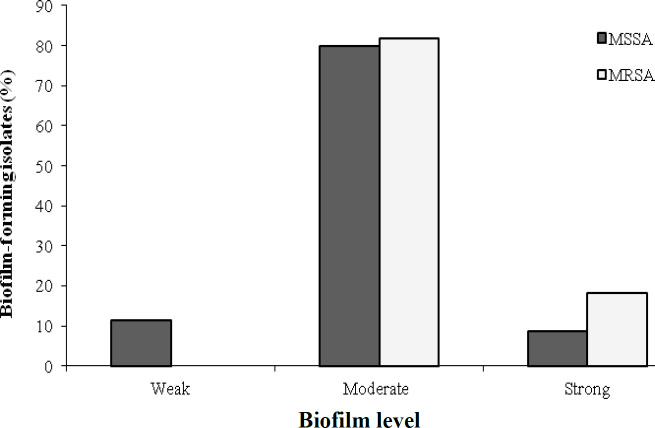
Frequency of biofilm formation among MSSA and MRSA isolates

The microtiter plate assay showed that all MSSA and MRSA isolates were capable of biofilm production. Strong biofilm producers represented 3 (8.6%) and 4 (18.1%) for MSSA and MRSA isolates, respectively, while moderate biofilm production for MSSA isolates was 28 (80%) in comparison with 18 (81.8%) for MRSA isolates. However, weak biofilm production was seen only in 4 (11.4%) MSSA isolates, but not in any MRSA isolates (Fig. 1). Our results showed no statistically significant differences between MSSA and MRSA isolates regarding weak, moderate and strong biofilm formation (*p *= 0.092; Fig. 1). 

**Antibiotic susceptibility test**

All *S. aureus *isolates were tested for their susceptibility against 14 commonly used antibiotics. All isolates were susceptible to vancomycin, while the antibiotic resistance rate of isolates for cefoxitin, benzylpenicillin, oxacillin, imipenem, tetracycline, erythromycin, clindamycin, and trimethoprim-sulfamethoxazole was as follows: 36.8% (21/57), 93% (53/57), 42.1% (24/57), 33.3% (19/57), 17.5% (10/57), 33.3% (19/57), 28.1% (16/57), and 10.5% (6/57), respectively. On the other hand, the antibiotic resistance rate for the following antibiotics: ciprofloxacin, gentamicin, tobramycin, fusidic acid, and rifampicin was 1.8% (1/57). The detailed pattern of antibiotic susceptibility and the level of biofilm production for MSSA and MRSA isolates are shown in Table 3. The MRSA isolates showed a statistically significant resistance pattern against the following antibiotics: cefoxitin, oxacillin, imipenem, tetracycline, clindamycin, and trimethoprim-sulfamethoxazole compared to MSSA (*p *< 0.05; Table 4). In addition, comparison of antibiotic resistance rates between the medical students and the patients has demonstrated no statistically significant differences between both groups (Table 4).

**Frequency of** ***ica*** **and MSCRAMMs-encoding genes**

**Fig. 2:**
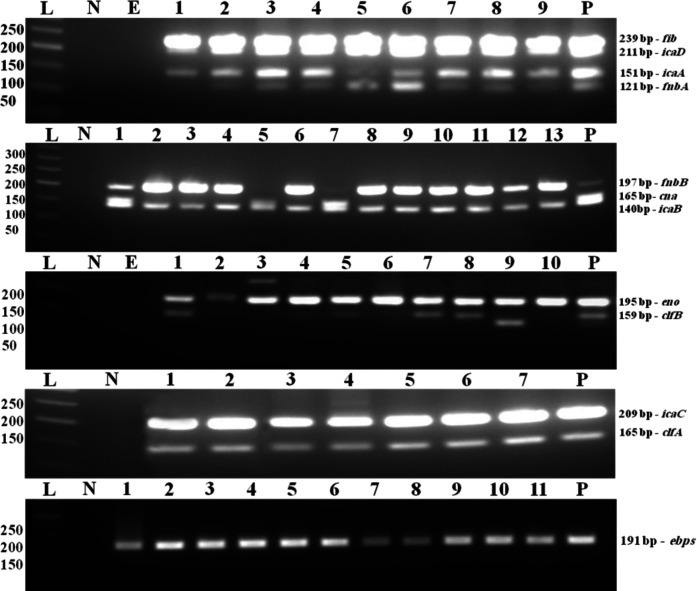
Multiplex PCR amplification products by agarose gel electrophoresis for the *ica* and MSCRAMMs-encoding genes. L, DNA molecular size marker (50 bp ladder); N, negative control; lanes 1-13, participants samples; P, positive control

The frequency of *ica* genes and MSCRAMMs-encoding biofilm genes was identified using multiple multiplex PCR reactions (Fig. 2). The first set included *icaA*, *icaD*, *fnbA*, and *fib*, and the PCR product sizes of each gene were 151, 211, 121, and 239 bp, respectively. The second reactions were comprised of *icaB*, *fnbB*, and *cna* with 140, 197, and 165 bp, respectively. The third set consisted of *clfB* and *eno* with 159 and 195 bp product size, respectively. The last multiplex set included *clfA* and *icaC* biofilm genes with sizes 165 and 209 bp, respectively. The gene *ebps* was run in a single PCR reaction with a product size of 191 bp (Fig. 2). The frequency of the *ica *genes and MSCRAMMs-encoding biofilm genes in MSSA and in MRSA isolates are detailed in Table 5. No significant statistical difference was noted between MSSA and MRSA regarding the frequency of the *ica* and the MSCRAMMs biofilm-forming genes. Also, further evaluation of the frequency of the biofilm forming genes between medical students and patients demonstrated no statistical significance (Table 6). 

**Table 3:** Antibiotic susceptibility pattern among MSSA and MRSA isolates in the biofilm-forming species

**Antibiotic**	**MSSA (N = 35)**					**MRSA (N = 22)**			
	**Weak biofilm** **N = 4**	**Moderate biofilm** **N = 28**	**Strong biofilm** **N = 3**	**Total** **N = 35**		**Moderate biofilm** **N = 18**	**Strong biofilm** **N = 4**	**Total** **N = 22**	
	**SN (%)**	**SN (%)**	**SN (%)**	**SN (%)**	**RN (%)**	**SN (%)**	**SN (%)**	**SN (%)**	**RN (%)**
Vancomycin	4 (11.4)	28 (80.0)	3 (8.6)	35 (100)	0	18 (81.8)	4 (18.2)	22 (100)	0
Cefoxitin	3 (8.6)	25 (71.4)	1 (2.9)	29 (82.9)	6 (17.1)	6 (27.3)	1 (4.5)	7 (31.8)	15 (68.2)
Benzyl penicillin	0	4 (11.4)	0	4 (11.4)	31 (88.6)	0	0	0	22 (100)
Oxacillin	4 (11.4)	27 (77.1)	2 (5.6)	33 (94.4)	2 (5.6)	0	0	0	22 (100)
Imipenem	4 (11.4)	27 (77.1)	3 (8.6)	34 (97.1)	1 (2.9)	3 (13.6)	1 (4.5)	4 (18.2)	18 (81.8)
Tetracycline	4 (11.4)	25 (71.4)	3 (8.6)	32 (91.4)	3 (8.6)	12 (54.6)	3 (13.6)	15 (68.2)	7 (31.8)
Erythromycin	4 (11.4)	20 (57.1)	2 (5.6)	26 (74.3)	9 (25.7)	11 (50)	1 (4.5)	12 (54.6)	10 (45.4)
Clindamycin	4 (11.4)	22 (62.9)	3 (8.6)	29 (82.9)	6 (17.1)	11 (50)	1 (4.5)	12 (54.6)	10 (45.4)
Ciprofloxacin	4 (11.4)	28 (80.0)	2 (5.6)	34 (97.1)	1 (2.9)	18 (81.8)	4 (18.2)	22 (100)	0
Trimethoprim-Sulfamethoxazole	4 (11.4)	28 (80)	2 (5.6)	34 (97.1)	1 (2.9)	14 (63.6)	3 (13.6)	17 (77.3)	5 (22.7)
									
Gentamycin	4 (11.4)	28 (80)	3 (8.6)	35 (100)	0	18 (81.8)	3 (13.6)	21 (95.5)	1 (4.5)
Tobramycin	4 (11.4)	28 (80)	3 (8.6)	35 (100)	0	18 (81.8)	3 (13.6)	21 (95.5)	1 (4.5)
Fusidic acid	4 (11.4)	28 (80)	3 (8.6)	35 (100)	0	18 (81.8)	3 (13.6)	21 (95.5)	1 (4.5)
Rifampicin	4 (11.4)	28 (80)	3 (8.6)	35 (100)	0	18 (81.8)	3 (13.6)	21 (95.5)	1 (4.5)

S, sensitive; R, resistant

**Table 4:** Antibiotic resistance pattern among students and patients in MRSA and MSSA nasal isolates

**Antibiotic**	**Participant category and bacterial type**							**Bacterial type**				
	**MRSA (N = 22)**				**MSSA (N = 35)**			**MRSA (N = 22)**			**MSSA (N = 35)**	
	**Students** **N (%)**	**Patients** **N (%)**	***p*** **value**		**Students** **N (%)**	**Patients** **N (%)**	***p*** ** value**	**Students** **N (%)**	***p*** **value**		**Patients** **N (%)**	***p*** **value**
Cefoxitin	7 (31.8)	8 (36.4)	0.616		5 (14.3)	1(2.9)	0.209	15 (68.2)	0.001		6 (17.1)	0.001
Benzylpenicillin	10 (45.5)	12 (54.5)	NA		20 (57.1)	11 (31.4)	0.165	22 (100)	0.100		31 (88.6)	0.10
Oxacillin	10 (45.5)	12 (54.5)	NA		2 (5.7)	0 (0)	0.353	22 (100)	0.001		2 (5.7)	0.001
Imipenem	8 (36.4)	10 (45.5)	0.632		0 (0)	1 (2.9)	0.400	18 (81.8)	0.001		1 (2.9)	0.001
Tetracycline	3 (13.6)	4 (18.2)	0.616		0 (0)	3 (8.6)	0.056	7 (31.8)	0.025		3 (8.6)	0.025
Erythromycin	3 (13.6)	7 (31.8)	0.185		6 (17.1)	3 (8.6)	0.474	10 (45.5)	0.124		9 (25.7)	0.124
Clindamycin	3 (13.6)	7 (31.8)	0.185		3 (8.6)	3 (8.6)	0.456	10 (45.5)	0.021		6 (17.1)	0.021
Ciprofloxacin	0 (0)	0 (0)	NA		1 (2.9)	0 (0)	0.600	0 (0)	0.424		1 (2.9)	0.424
Trimethoprim- Sulfamethoxazole	1 (4.5)	4 (18.2)	0.218		1 (2.9)	0 (0)	0.600	5 (27.7)	0.027		1 (2.9)	0.017
Gentamycin	0 (0)	1 (4.5)	0.545		0 (0)	0 (0)	NA	1 (4.5)	0.203		0 (0)	0.203
Tobramycin	0 (0)	1 (4.5)	0.545		0 (0)	0 (0)	NA	1 (4.5)	0.203		0 (0)	0.203
Fusidic acid	0 (0)	1 (4.5)	0.545		0 (0)	0 (0)		1 (4.5)	0.203			0.203
Rifampicin	0 (0)	1 (4.5)	0.545		0 (0)	0 (0)		1 (4.5)	0.203			0.203

## DISCUSSION

Staphylococcal infections in the hospital setting represent a huge burden globally^[^^21^^]^. The emergence of multidrug-resistant strains has rendered the antimicrobial approach ineffective against staphylococcal infections. Biofilm formation by *S. aureus* has an important role in pathogenesis, immune evasion, and antibiotic resistance, hence studying the ability of *staphylococcus *isolates to form biofilm will help to determine their severity and virulence^[^^22^^]^. 

In addition to its global impact, the development of resistant *S. aureus* strains is of particular importance in Jordan due to high rates of antimicrobial resistance emerged largely by antibiotic misuse practices and the recent increase in refugee (predominantly from Syria) numbers amongst whom the prevalence of resistant MRSA strains is relatively high^[^^23^^,^^24^^]^. These geopolitical events can largely affect the distribution of antibiotic-resistant bacteria, and a continuous assessment of infection, colonization, and antibiotic sensitivity profiles is required^[^^25^^]^.

While previous studies, including our own, have looked into antibiotic resistance patterns of *S. aureus* species isolated from different subpopulations in Jordan, they did not examine the underlying virulence mechanisms, specifically biofilm formation^[^^15^^,^^26^^]^. Accordingly, in this work, we attempted to provide a more recent analysis of MRSA and MSSA strains in carriers as well as to investigate the association of drug-resistance with biofilm-forming genes. Hence, we have selected two populations of study that are susceptible to the acquisition of staphylococcal infection. The two groups were comprised of patients admitted into four different medical care wards and medical students under training in the same facility.

In this study, biofilm formation among the *S. aureus* isolates obtained from the nasal mucosa of patients and medical students were determined phenotypically using two methods, the microtiter plate assay and the CRA method. Expectedly, all the 57 isolates of *S. aureus* from both patients and students were able to produce biofilm using the microtiter plate assay. Strong, moderate and weak biofilm formation was observed in 7 (12.3%), 46 (80.7%), and 4 (7%) of *S. aureus* isolates (Fig. 1). However, the CRA method suggested that only 45 (79%) of the *S. aureus* isolates were capable of biofilm production. These data indicate that the microtiter plate assay is more sensitive than CRA in determining biofilm production. This finding is in agreement with other studies that compared the sensitivity of phenotypic biofilm production^[^^27^^,^^28^^]^.

**Table 5:** Comparison of the frequency of the biofilm-forming genes between MSSA and MRSA isolates

**Biofilm gene**	**MSSA (N = 35)**		**MRSA (N = 22)**		***p *** **value**
	**Negative** **N (%)**	**Positive** **N (%)**	**Negative** **N (%)**	**Positive** **N (%)**	
*icaA*	8 (22.9)	27 (77.1)	3 (13.6)	19 (86.4)	0.390
*icaD*	1 (2.9)	34 (97.1)	0 (0)	22 (100)	0.424
*icaC*	1 (2.9)	34 (97.1)	0 (0)	22 (100)	0.424
*icaB*	2 (5.7)	33 (94.3)	0 (0)	22 (100)	0.254
*fnbA*	10 (28.6)	25 (71.4)	4 (18.2)	18 (81.8)	0.375
*fnbB*	5 (14.3)	30 (85.7)	2 (9.1)	20 (90.9)	0.561
*fib*	4 (11.4)	31 (88.6)	2 (9.1)	20 (90.9)	0.780
*eno*	0 (0)	35 (100)	0 (0)	22 (100)	NA
*cna*	23 (65.7)	12 (34.3)	13 (59.1)	9 (40.9)	0.614
*ebps*	2 (5.7)	33 (94.3)	0 (0)	22 (100)	0.254
*clfA*	3 (8.6)	32 (91.4)	1 (4.5)	21 (95.5)	0.562
*clfB*	9 (25.7)	26 (74.3)	3 (13.6)	19 (86.4)	0.276

Interestingly, we failed to record a statistically significant difference in biofilm formation between MSSA and MRSA isolates in both the patient and medical student groups. These results disclose that MSSA strains isolated in our work have almost equal nosocomial infection potential to MRSA, which explains in part the higher rate of MSSA infection in both studied groups. 

Biofilm formation is considered one of the major virulence factors in antibiotic-resistant bacteria, especially MRSA. For a better understanding of the complex process of biofilm production in Staphylococcal infections, further phenotypic and genotypic characterization of the *ica* locus genes is needed^[^^29^^]^. Expression of the *ica* genes was regulated by other genes such as *agr*, *sigB*, and *sar*, which interact with each other to regulate biofilm production^[^^30^^]^. Also, an increasing number of different adhesion molecules in *S. aureus* has been established^[^^31^^]^. 

In the current survey, a clear association was found between the biofilm formation and the presence of the* icaD*, where all MRSA and 97.1% of the MSSA isolates harboring this gene produced biofilm. The prevalence of the *icaD* gene in MRSA and MSSA isolates showed no statistical significance. In agreement with our findings, other studies have suggested a similar relation between the prevalence of *icaD *gene and phenotypic biofilm formation^[^^32^^,^^33^^]^, whereas some studies have revealed that the presence of the *icaD *gene is not necessarily associated with biofilm formation^[^^34^^]^. 

The *fnbA* and *fnbB* genes play a role in facilitating adhesion and invasion of bacteria, and *fnbB *presence is correlated with biofilm formation and virulence in the human host^[^^35^^]^. In our study, *fnbA* and *fnbB *gene prevalence in MRSA isolates was determined to be 81.8% and 90.9%, respectively. Similarly, high *fnbA* and *fnbB *gene occurrences were reported in studies by Gowrishankar *et al*.^[^^36^^]^ in 2016 and Mirzaee *et al*.^[^^37^^]^ in 2015. Likewise, *fnbA* and *fnbB *gene prevalence were 71.4% and 85.7% in MSSA isolates, respectively. The *fib* gene was detected in almost 90% of the isolates in both MSSA and MRSA isolates. The *fib*, in addition to its role in binding to fibrinogen, can interrupt platelet aggregation and interfere with complement cascade activation within the host^[^^38^^]^. These actions collectively render the *fib* an important virulence factor in Staphylococcal infections.

The clumping factor plays an important role in colonization and attachment of *S. aureus* to the epithelium and skin surfaces. In this study, the *clfA *and *clfB *prevalence were 95.5% and 86.4% in MRSA and MSSA isolates, respectively. The results of other investigations exhibited a similarly high prevalence among these genes^[^^39^^,^^40^^]^. The c*na* is another important virulence factor in *S. aureus* infections, which binds to collagen^[^^41^^]^. In agreement with other studies^[^^42^^,^^43^^]^, the incidence of the *cna* gene in our study among the MRSA isolates was 40.9%. The *ebps* gene, which mediates the binding of *S. aureus* to elastin^[^^44^^]^, was found in all MRSA isolates and 94% of the MSSA isolates. Likewise, all MRSA and MSSA isolates in this study had expressed the *eno* gene, this was in concordance with other studies conducted^[^^42^^]^.

**Table 6:** Comparison of the frequency of MRSA and MSSA isolates and percentages of positive biofilm-forming genes between students and patients

**Characteristic**	**MD students** **N (%)**	**Patients** **N (%)**	**X²**	***p*** **value**
MRSA	10 (32.3)	12 (46.2)	1.152	0.283
MSSA	21(67.7)	14 (53.8)	1.152	0.283
*icaA*	26 (83.9)	20 (76.9)	0.438	0.508
*icaB*	30 (96.8)	25 (96.2)	0.016	0.899
*icaC*	31 (100.0)	25 (96.2)	1.214	0.271
*icaD*	31 (100.0)	25 (96.2)	1.214	0.271
*fnbA*	22 (71.0)	21 (80.8)	0.733	0.392
*fnbB*	27 (87.1)	18 (69.2)	2.716	0.099
*fib*	26 (83.9)	23 (88.5)	0.247	0.619
*cna*	11 (35.5)	10 (38.5)	0.054	0.816
*ebps*	30 (96.8)	25 (96.2)	0.016	0.899
*clfA*	3 (9.7)	3 (11.5)	0.052	0.820
*clfB*	15 (48.4)	10 (38.5)	0.566	0.452
*eno*	31 (100)	26 (100)	NA	NA
				
Total	31 (54.4)	26 (45.6)		

Finally, we analyzed the antimicrobial susceptibility of both MSSA and MRSA isolates from both groups. Our aim was to investigate whether there was a significantly higher rate of staphylococcal resistance to a number of commonly used antibiotics in the patient group in comparison to the student group, especially since the former group showed a higher rate of previous antibiotic use and hospitalization. Of the antibiotics tested, vancomycin is generally considered the last resort for *S. aureus *eradication, and it is completely effective in combating these infections, though certain studies have shown reduced susceptibility^[^^45^^]^. In our study, all *S. aureus* isolates were susceptible to vancomycin and similarly, most isolates were susceptible to ciprofloxacin, gentamicin, tobramycin, fusidic acid, and rifampicin. No significant differences in antibiotic resistance were recorded between patients’ and students’ isolates, indicating that previous antibiotic use or hospitalization did not majorly contribute to antimicrobial resistance in the studied populations. However, and as expected, MRSA isolates from both patients and medical students were more resistant than MSSA to cefoxitin, oxacillin, imipenem, tetracycline, clindamycin, and trimethoprim-sulfamethoxazole, which supports former observations^[^^46^^]^.

While the population size was sufficient to draw the previously stated conclusions, it is noteworthy that it also represents a limitation to further interpretation of the data. Furthermore, the two studied populations were either admitted or attending the same healthcare institution, which does not optimally represent the status of staphylococcal infection rate in the country or the profile of antibiotic resistance. In our future studies, we plan on recruiting a larger, more representative population from several, geographically distinct healthcare facilities across the country.

Our findings showed a significantly higher resistance pattern in the MRSA isolates in comparison to MSSA against six antibiotics (cefoxitin, oxacillin, imipenem, tetracycline, clindamycin, and trimethoprim-sulfa-methoxazole). However, there was no significant difference in the biofilm formation or the expression of its related genes between the two staphylococcal strains. Additionally, we did not record significant differences in antibiotic resistance or biofilm formation in staphylococcal strains isolated from patients and medical students. Lastly, the carrier rate of *S.* aureus amongst the studied populations was 11.6%. Implementation of strict hospital infection control measures would further reduce the spread of infection from carriers to patients or medical trainees in hospital wards. 

## References

[1] Laux C, Peschel A, Krismer B (2019). Staphylococcus aureus colonization of the human nose and interaction with other microbiome members. Microbiol spectrum.

[2] Anderson MJ, Schaaf E, Breshears LM, Wallis HW, Johnson JR, Tkaczyk C, Sellman BR, Sun J, Peterson ML (2018). Alpha-Toxin contributes to biofilm formation among staphylococcus aureus wound isolates. Toxins (Basel).

[3] Shrestha LB, Baral R, Khanal B (2019). Comparative study of antimicrobial resistance and biofilm formation among Gram-positive uropathogens isolated from community-acquired urinary tract infections and catheter-associated urinary tract infections. Infectiont drug resistance.

[4] Desrousseaux C, Sautou V, Descamps S, Traore O (2013). Modification of the surfaces of medical devices to prevent microbial adhesion and biofilm formation. Journal of hospital infection.

[5] Kirui DK, Weber G, Talackine J, Millenbaugh NJ (2019). Targeted laser therapy synergistically enhances  efficacy of antibiotics against multi-drug resistant staphylococcus aureus and pseudomonas aeruginosa biofilms. Nanomedicine.

[6] Upreti N, Rayamajhee B, Sherchan SP, Choudhari MK, Banjara MR (2018). Prevalence of methicillin resistant Staphylococcus aureus, multidrug resistant and extended spectrum β-lactamase producing Gram negative bacilli causing wound infections at a tertiary care hospital of Nepal. Antimicrobial resistance and infection control.

[7] Otto M (2018). Staphylococcal biofilms. Microbiolgy spectrum.

[8] Lerch MF, Schoenfelder SMK, Marincola G, Wencker FDR, Eckart M, Forstner KU, Sharma CM, Thormann KM, Kucklick M, Engelmann S, Ziebuhr W (2019). A non-coding RNA from the intercellular adhesion (ica) locus of Staphylococcus epidermidis controls polysaccharide intercellular adhesion (PIA)-mediated biofilm formation. Molecular microbiology.

[9] Mirzaee M, Najar-Peerayeh S, Behmanesh M, Moghadam MF (2015). Relationship between adhesin genes and biofilm formation in vancomycin-intermediate Staphylococcus aureus clinical isolates. Current microbiolgy.

[10] Archer NK, Mazaitis MJ, Costerton JW, Leid JG, Powers ME, Shirtliff ME (2011). Staphylococcus aureus biofilms: properties, regulation, and roles in human disease. Virulence.

[11] Arciola CR, Campoccia D, Ravaioli S, Montanaro L (2015). Polysaccharide intercellular adhesin in biofilm: structural and regulatory aspects. Frontiers in cellular and infection microbiolgy.

[12] Morales M, Mendez-Alvarez S, Martin-Lopez JV, Marrero C, Freytes CO (2004). Biofilm: the microbial "bunker" for intravascular catheter-related infection. Support care cancer.

[13] Ko YP, Flick MJ (2016). Fibrinogen is at the interface of host defense and pathogen virulence in Staphylococcus aureus infection. Seminars in thrombosis and hemostasis.

[14] Theos KR, Johnson KM, Johnson DW (2019). Staphylococcus aureus antibiotic susceptibilities in infections in an outpatient dermatology office on O'ahu. Hawaii journal medicine public health.

[15] Al-Tamimi M, Himsawi N, Abu-Raideh J, Abu Jazar D, Al-Jawaldeh H, Al Haj Mahmoud S, Hijjawi N, Hawamdeh H (2018). Nasal colonization by methicillin-sensitive and methicillin-resistant. Staphylococcus aureus among medical students. The journal of infection in developing countries.

[16] Magana M, Sereti C, Ioannidis A, Mitchell CA, Ball AR, Magiorkinis E, Chatzipanagiotou S, Hamblin MR, Hadjifrangiskou M, Tegos GP (2018). Options and limitations in clinical investigation of bacterial biofilms. Clinical microbiol reviews.

[17] Nourbakhsh F, Namvar AE (2016). Detection of genes involved in biofilm formation in Staphylococcus aureus isolates. GMS Hygiene and infection control.

[18] Breves A, Miranda CAC, Flores C, Filippis Id, Clementino MM (2015). Methicillin and vancomycin resistant Staphylococcus aureus in health care workers and medical devices. Jornal brasileiro de patologia e medicina laboratorial.

[19] Atshan SS, Nor Shamsudin M, Sekawi Z, Lung LT, Hamat RA, Karunanidhi A, Mateg Ali A, Ghaznavi-Rad E, Ghasemzadeh-Moghaddam H, Chong Seng JS, Nathan JJ, Pei CP (2012). Prevalence of adhesion and regulation of biofilm-related genes in different clones  of Staphylococcus aureus. Journal biomedicine biotechnolgy.

[20] de Castro Melo P, Ferreira LM, Filho AN, Zafalon LF, Vicente HI, de Souza V (2013). Comparison of methods for the detection of biofilm formation by Staphylococcus aureus isolated from bovine subclinical mastitis. Brazilian journal of microbiology.

[21] Grundmann H, Aires-de-Sousa M, Boyce J, Tiemersma E (2006). Emergence and resurgence of meticillin resistant Staphylococcus aureus as a public health threat. The lancet.

[22] Savage VJ, Chopra I, Neill AJ (2013). Staphylococcus aureus biofilms promote horizontal transfer of antibiotic resistance. Antimicrobial agents and chemotherapy.

[23] Kossow A, Stühmer B, Schaumburg F, Becker K, Glatz B, Möllers M, Kampmeier S, Mellmann A (2018). High prevalence of MRSA and multi-resistant gram-negative bacteria in refugees admitted to the hospital-But no hint of transmission. PLoS one.

[24] Aro T, Kantele A (2018). High rates of meticillin resistant Staphylococcusaureus among asylum seekers and refugees admitted to Helsinki University Hospital, 2010 to 2017. Eurosurveillance.

[25] Stefani S, Chung DR, Lindsay JA, Friedrich AW, Kearns AM, Westh H, MacKenzie FM (2012). Meticillin-resistant Staphylococcusaureus (MRSA): global epidemiology and harmonisation of typing methods. International journal of antimicrobial agents.

[26] Al-Zoubi MS, Al-Tayyar IA, Hussein E, Jabali AA, Khudairat S (2015). Antimicrobial susceptibility pattern of Staphylococcus aureus isolated from clinical specimens in northern area of jordan. Iranian journal of microbiolgy.

[27] Torlak E, Korkut E, Uncu AT, Şener Y (2017). Biofilm formation by Staphylococcus aureus isolates from a dental clinic in Konya, Turkey. Journal of infection and public health.

[28] Triveni AG, Suresh Kumar M, Manjunath C, Shivannavar CT, Gaddad SM (2018). Biofilm formation by clinically isolated Staphylococcus aureus from India. The journal of infection in developing countries.

[29] Darwish SF, Asfour HA (2013). Investigation of biofilm forming ability in Staphylococci causing bovine mastitis using phenotypic and genotypic assays. Scientific world journal.

[30] Bischoff M, Entenza JM, Giachino P (2001). Influence of a functional sigB operon on the global regulators sar and agr in staphylococcus aureus. Journal of bacteriology.

[31] Foster TJ, Geoghegan JA, Ganesh VK, Höök M (2014). Adhesion, invasion and evasion: the many functions of the surface proteins of Staphylococcus aureus. Nature reviews microbiology.

[32] Serray B, Oufrid S, Hannaoui I, Bourjilate F, Soraa N, Mliji M, Sobh M, Hammoumi A, Timinouni M, El Azhari M (2016). Genes encoding adhesion factors and biofilm formation in methicillin-resistant Staphylococcus aureus in Morocco. The journal of infection in developing countries.

[33] Liberto MC, Matera G, Quirino A, Lamberti AG, Capicotto R, Puccio R, Barreca GS, Focà E, Cascio A, Focà A (2009). Phenotypic and genotypic evaluation of slime production by conventional and molecular microbiological techniques. Microbiological research.

[34] Růžička F, Holá V, Votava M, Tejkalová R, Horvát R, Heroldová M, Woznicová V (2004). Biofilm detection and the clinical significance of Staphylococcus epidermidis isolates. Folia microbiologica.

[35] Cha JO, Yoo JI, Yoo JS, Chung H-S, Park SH, Kim HS, Lee YS, Chung GT (2013). Investigation of biofilm formation and its association with the molecular and clinical characteristics of methicillin-resistant Staphylococcus aureus. Osong public health and research perspectives.

[36] Gowrishankar S, Kamaladevi A, Balamurugan K, Pandian SK (2016). In vitro and in vivo biofilm characterization of Methicillin resistant Staphylococcus aureus from patients associated with pharyngitis infection. BioMed research iternational.

[37] Mirzaee M, Najar-Peerayeh S, Behmanesh M (2015). Prevalence of fibronectin binding protein (FnbA and FnbB) genes among clinical isolates of methicillin resistant Staphylococcus aureus. Molecular genetics, microbiology and virology.

[38] Posner MG, Upadhyay A, Abubaker AA, Fortunato TM, Vara D, Canobbio I, Bagby S, Pula G (2016). Extracellular Fibrinogen-binding protein (Efb) from Staphylococcus aureus inhibits the formation of platelet leukocyte complexes. Journal biological chemistry.

[39] Klein RC, Fabres-Klein MH, Brito MA, Fietto LG, Ribon Ade O (2012). Staphylococcus aureus of bovine origin: genetic diversity, prevalence and the expression of adhesin-encoding genes. Veterinary microbiology.

[40] Ghasemian A, Najar Peerayeh S, Bakhshi B, Mirzaee M High Frequency of icaAD, clumping factors A/B, fib and eno Genes in Staphylococcus aureus Species Isolated From Wounds in Tehran, Iran during 2012-2013. Archive of clinical infectious diseases.

[41] Madani A, Garakani K, Mofrad MRK (2017). Molecular mechanics of Staphylococcus aureus adhesin, CNA, and the inhibition of bacterial adhesion by stretching collagen. PLoS one.

[42] Dai J, Wu S, Huang J, Wu Q, Zhang F, Zhang J, Wang J, Ding Y, Zhang S, Yang X, Lei T, Xue L, Wu H (2019). prevalence and characterization of Staphylococcus aureus isolated From pasteurized milk in China. Frontiers in microbiology.

[43] Kouidhi B, Zmantar T, Hentati H, Bakhrouf A (2010). Cell surface hydrophobicity, biofilm formation, adhesives properties and molecular detection of adhesins genes in Staphylococcus aureus associated to dental caries. Microbial pathogenesis.

[44] Nakakido M, Tanaka Y, Tsumoto K (2007). The N-terminal domain of elastin-binding protein of Staphylococcusaureus changes its secondary structure in a membrane-mimetic environment. The journal of biochemistry.

[45] Shekarabi M, Hajikhani B, Salimi Chirani A, Fazeli M, Goudarzi M (2017). Molecular characterization of vancomycin-resistant Staphylococcus aureus strains isolated from clinical samples: A three year study in Tehran, Iran. PLoS one.

[46] Chen Z, Han C, Huang X, Liu Y, Guo D, Ye X (2018). A molecular epidemiological study of methicillin-resistant and methicillin-susceptible Staphylococcus aureus contamination in the airport environment. Infection drug resistance.

